# Supplementary material for: Numerical investigation of seismic amplification characteristics in loess ridge region of Xiji, northwest China
Source: PLoS One. 2024 Feb 5;19(2):e0297287. doi: 10.1371/journal.pone.0297287 (PMC10843532; doi:10.1371/journal.pone.0297287)
Supplement: Attachment — Submitted filename: Response to Reviewers.docx [file pone.0297287.s002.docx]

Response to the Review Comments

We would like to thank you for your careful reading, helpful comments, and constructive suggestions, which has significantly improved the presentation of our manuscript.

We have carefully considered all comments from the reviewers and revised our manuscript accordingly. The manuscript has also been double-checked, and the typos and grammar errors we found have been corrected. In the following section, we summarize our responses to each comment from the reviewers. We believe that our responses have well addressed all concerns from the reviewers. Many thanks to the reviewer for providing valuable feedback, especially for guiding us in our future research directions.

We hope our revised manuscript can be accepted for publication. The response as shown in below.

1. in page 4, please provide more information about the Loess Plateau region. Where it is, what it is, etc, as this information is not known to the reader. Probably this is well known in China, but the target audience of the journal is worldwide so this needs to be explained further and in detail.

Response1: We thank the reviewer for pointing out this issue. We have provided more information about the Loess Plateau region and added a map about the location (Fig 1).

“The Loess Plateau is located in the mid to upper reaches of the Yellow River in northwest China, primarily encompassing provinces such as Gansu, Shaanxi, Shanxi, and the Ningxia Hui Autonomous Region. It spans an area of approximately 630,000 km^2^, characterized by an overall topography of higher elevations in the west gradually descending to lower elevations in the southeast. The average elevation decreases from around 2000 m in the west to 1000 m in the southeast. In this region, a unique climate has led to the widespread distribution of extensive, continuous, and thick loess deposits. Over the past two million years, the interplay of loess accumulation, erosion, and transportation, influenced by both internal and external geological forces, has given rise to the distinctive modern landforms of the Loess Plateau, including loess tableland, loess ridges, and loess hills.”

Fig .1 The location of Loess Plateau

2. a general comment is about the generalization of the results: How applicable are the findings to other loess regions with varying soil properties? How can the study's findings be generalized to other geographic regions with loess formations? Are there specific soil properties or environmental conditions where these findings might not be applicable?

Response2: The generalization of the study's findings to other loess regions with varying soil properties is a crucial aspect to consider. While the research provides valuable insights into seismic effects on loess ridge terrain, the applicability of the findings to different geographic regions with diverse soil properties needs careful examination.

The loess ridges in different regions of the Loess Plateau exhibit significant variations in topography and substantial differences in loess thickness. Moreover, the physical and mechanical properties of loess vary significantly among different regions, introducing uncertainties into the seismic amplification effects on loess ridges. Delving into the impact of these factors is a formidable task.

In this study, the model focused on the south of Ningxia Autonomous Region and east of Gansu Province, a seismic-prone area in the western Loess Plateau. The loess in this region demonstrates uniform thickness, with relatively minor variations in physical and mechanical properties, making the results applicable to the approximately 10,000 km^2^ of this region.

To ensure the specificity of the paper and the rigor of its conclusions, we considered the applicability of the findings. As a result, we modified the title of the paper to " Numerical Investigation of Seismic Amplification Characteristics in Loess Ridge Region of Xiji, Northwestern China " and made corresponding adjustments to the paper. We appreciate the reviewer for pointing out the direction for further work. In the future, we will conduct an in-depth study of the seismic amplification effects on loess topography based on different regions and landform units, providing applicable results for earthquake-resistant design in the Loess Plateau region.

3. Frequency Range Analysis: You mention that the frequency (f) of the seismic input time history is taken at 1 Hz, 2 Hz, 5 Hz, and 10 Hz. Is there a specific reason for choosing the specific frequency range used in the study? What was the scientific basis for choosing the specific frequency range used in this study? How do different frequency ranges influence the seismic amplification observed in loess slopes?

Reponse3: In this study, the selection of specific frequency ranges is typically driven by the consideration of understanding the amplification effects of different seismic waves on various loess ridge terrains. The choice of this specific frequency range arises from considerations of real seismic events and the needs of seismic input analysis for structural engineering. The seismic spectrum encompasses a wide range, with the common spectrum being in the 0.1-10 Hz range, which is also of interest in seismic input for structural analysis. Taking into account computational efficiency, model size, quantitative analysis difficulty, mesh element size, and previous relevant research literature, frequencies of 1 Hz, 2 Hz, 5 Hz, and 10 Hz were chosen as specific inputs.

A brief summary of the research results is as follows: Under low-intensity seismic motion at the bottom (PGA=0.1 m/s²), the amplification factor ε relative to the slope toe, instead of being uniformly greater than 1 or increasing with slope height (η), exhibits a significant nonlinear characteristic. The ε-η relationship shows rhythmic amplification and reduction, becoming more frequent with higher frequencies and taller slopes. At the top of the loess ridge, ε gradually decreases with increasing frequency. At 1 Hz, 2 Hz, and 5 Hz frequencies, ε shows a relatively clear linear growth relationship with the crest height, while at 10 Hz, ε does not exhibit a significant variation with crest height and generally remains below 1, indicating a trend of attenuation.

In contrast, under high-intensity seismic motion at the bottom (PGA=1 m/s²), at 1 Hz and 2 Hz, the ε-η trend on the slope surface is generally consistent, with ε values generally higher than those in the low-intensity scenario. For 5 Hz and 10 Hz strong seismic motions, compared to the same-frequency small seismic motions, a more pronounced rhythmic pattern is observed on the lower and shorter loess slopes (H=50m, 60m). The ε-η trend is different, and for steeper slopes, the dispersion between maximum and minimum values of ε decreases. At the top of the loess ridge, ε decreases with increasing frequency, with ε values generally higher than those in the low-intensity seismic motion scenario, but ε does not show a linear relationship with crest height.

4. The authors could elaborate more on how each variable within the numerical model contributes to the overall seismic response. Specifically, understanding the individual impact of variables like soil density, cohesion, and internal friction angle would be beneficial. Additionally, how were these variables calibrated or adjusted to reflect real-world conditions more accurately?

Reponse4：Thank you for your question. In the numerical model, the constitutive model used is the Mohr-Coulomb model, and the parameters are derived from laboratory tests on undisturbed soil samples. In the dynamic response of the soil slope, the modulus and damping of the soil are crucial. In this study, hysteresis damping is a key consideration for describing the nonlinear hysteresis behavior of the soil under seismic action. Hysteresis damping plays a role in characterizing the nonlinearity and damping properties of loess soils in seismic engineering, aiding in a more accurate simulation of the actual dynamic response of the soil.

In our research, we conducted resonance column tests on loess samples at different depths (5m, 10m, 20m, 30m) to obtain the dynamic modulus and damping characteristics of the soil. The hysteresis damping was modeled using a three-parameter form, and through iterative adjustments, it was ensured to closely match the results of dynamic tests. This hysteresis damping model is material-independent and adopts the damping curve from dynamic tests, effectively capturing the dynamic nonlinearity of loess soils. This contributes to a more realistic simulation of the soil's dynamic response to seismic action, enhancing the accuracy and reliability of the numerical model.

5. How does the temporal aspect of seismic activity (e.g., duration and frequency of seismic events over time) influence the long-term stability of loess slopes? It would be insightful if the study included a temporal analysis to predict changes in slope stability over extended periods of seismic activity.

Reponse5：This study primarily focuses on analyzing the seismic amplification effects on loess ridges, specifically addressing the seismic input at the ridge top. In this research, no sliding occurred on any slope.

We appreciate your valuable insight into the crucial role of the duration and frequency of seismic activity in the dynamic stability of loess slopes. The study indicates that loess slopes undergo damage and cracking under seismic forces, as evidenced by observations such as the seismic damage caused by the 2013 Ms 6.6 Minxian earthquake in loess slopes, leading to subsequent instability and sliding during aftershocks (Wang et al, 2017, Loessial landslides induced by the Minxian–Zhangxian Ms6. 6 earthquake of China in 2013). Presently, field investigations, laboratory experiments, and numerical simulations have not quantitatively expressed the seismic damage characteristics of loess under different intensities, durations, and frequencies of seismic activity. Studying the long-term stability of loess slopes post-earthquake poses a challenging and crucial issue.

6. Considering the complexity of soil-structure interactions during seismic events, how might the presence of buildings or other structures on or near the loess slopes impact the findings? A deeper exploration into how structures modify the seismic wave propagation and amplification would add value to the study.

Reponse6：Thank you to the reviewer for bringing up another significant and broad research question. The presence of buildings or other structures on or near loess slopes could indeed have a substantial impact on the study's findings. Factors such as the mass, shape, and foundation characteristics of buildings may influence the dynamic response of adjacent soil. The interaction between soil and structures could alter the propagation path of seismic waves. Additionally, the location, density, and spacing of structures may also have complex effects on seismic response. These issues are highly deserving of further in-depth investigation.

7. The integration of historical seismic data into the numerical model could enhance the study's relevance. How might the incorporation of past seismic records from the study area refine the model's predictions? This approach could validate the model's accuracy in simulating real-world scenarios.

Reponse7：Thank you for the valuable suggestion. The incorporation of historical seismic data can authentically reflect the seismic characteristics of the slope site and validate the accuracy of numerical simulation results. However, in the study area, there is a lack of seismograph networks specifically for loess slope terrain, and there are no actual seismic records for loess slopes to compare with.

8. Loess soil properties can significantly change with varying water content. How would the presence of underground water or a varying water table affect the seismic response of loess slopes? A discussion on the impact of hydrological conditions would provide a more comprehensive understanding of the seismic behavior of loess soils.

Reponse8：This study utilized real soil data from the loess ridge site, representing the groundwater characteristics of the regional loess ridge slopes. The article presented the physical and dynamic parameters of loess at different depths, reflecting the varying influences of different water contents on soil properties. As mentioned by the reviewer, loess possesses unique structural characteristics and abundant soluble salts, contributing to its water sensitivity. Some studies suggest that saturated loess has the potential for liquefaction (Wang G et al, 2014, Pore-pressure generation and fluidization in a loess landslide triggered by the 1920 Haiyuan earthquake, China: a case study; Pei et al, 2017. Experimental case study of seismically induced loess liquefaction and landslide). Investigating the impact of groundwater level and its variations on the dynamic response of loess slopes requires detailed analysis of the slope's geological structure, hydrogeology, and soil properties.

9. Apart from seismic activities, loess slopes are also subjected to non-seismic ground movements such as landslides or erosion. How do these factors interplay with seismic forces in influencing slope stability? An analysis of the combined effects would provide a more holistic view of the risks associated with loess slopes.

Reponse9：Thank you once again for providing valuable research directions. The Loess Plateau is one of the areas in China severely affected by soil erosion. During our field surveys, we often observe erosion phenomena on slopes, such as loess sinkholes, gullies, and natural bridges. These erosional processes, occurring over the long term, may lead to progressive instability of loess slopes, contributing to the widespread development of landslides on the Loess Plateau (Peng, et al. 2019, Distribution and genetic types of loess landslides in China). If combined with sudden and intense earthquakes, these erosional processes can trigger numerous loess earthquake landslides, resulting in significant loss of life and property (Xu et al. Landslides of the 1920 Haiyuan earthquake, northern China). Research on the dynamic stability assessment and prediction of loess slopes considering non-seismic ground movement factors under strong seismic activity is a crucial issue urgently needing attention in the Loess Plateau region.

10. A sensitivity analysis of key parameters (e.g., slope height, soil properties, seismic intensity) could provide insights into their relative importance in the model. Which parameters are most sensitive, and how do changes in these parameters affect the overall seismic response?

Reponse10: We appreciate the reviewer’s clarification. In this study, sensitivity analysis was not conducted. The investigation focused on the seismic response of loess slopes under varying slope heights, two seismic intensities, and the influence of different seismic frequencies. Considering the suggestions from the reviewer, it is proposed that future work should include sensitivity analysis to identify which key parameters exhibit the highest sensitivity in the seismic response of loess slopes.

11. How would the seismic response of loess slopes compare with slopes made of different soil types, such as clay or sandy soils? A comparative study could highlight the unique aspects of loess soil behavior under seismic stress.

Reponse11：While it is true that a better understanding of the dynamic response of loess ridges can be gained by comparing them with other types of soil slopes, seismic response is not only related to soil type but also influenced by various factors such as slope geometry, subsurface structure, seismic characteristics, etc. It is challenging to find research cases similar to this study due to the unique characteristics of loess ridges. However, a basic comparison was made with the results of Paolucci's study (Paolucci, R, 2002, "Amplification of earthquake ground motion by steep topographic irregularities"). The findings in this study are somewhat similar to Paolucci's research on double-sided slopes, where the relative amplification at the slope top was observed to be in the range of 1.5-2 times.

12. Based on the findings of the study, what practical guidelines or recommendations can the authors provide for engineering applications, such as construction practices, slope reinforcement techniques, and early warning systems? Detailed suggestions would make the study more applicable to engineers and urban planners working in loess regions. How can the findings of this study be translated into practical applications for disaster planning and risk mitigation in regions with loess formations? Are there specific strategies or tools that the authors would recommend based on their research?

Reponse12：Based on the findings of the study, compared to the horizontal seismic impact coefficient range of 1.1 to 1.6 proposed by the Chinese "Code for Seismic Design of Buildings" (GB5011-2010, 2016 edition) for seismic vulnerable areas such as protruding mountain ridges, we recommend that the horizontal seismic impact coefficient at the top of loess ridges should be multiplied by a range of 1.5 to 2.2. This adjustment takes into account the amplification effects of loess slopes without considering the influence of slope height.

Additionally, considering the rhythmic changes in slope amplification effects with slope height and frequency, where higher slopes exhibit more frequent rhythmic changes, calculating the horizontal seismic impact coefficient for the dynamic stability of loess ridge slopes should account for such variations. This approach facilitates better engineering design and the assessment of slope instability positions.

13. The authors are requested to provide a comprehensive explanation of the criteria and rationale behind the selection of the specific numerical models used in this study. How do these models compare with other available models in terms of accuracy, reliability, and relevance to the seismic behavior of loess slopes?

Reponse13：When establishing the numerical model, we based it on two crucial elements to ensure its applicability to the study of seismic amplification effects in loess ridges:

1. Authenticity and Applicability: The selected loess ridge site was chosen for its authenticity, representing the topography and dynamic characteristics of loess ridges. The model's soil layers needed to accurately reflect the regional geological structure, and the model's dimensions needed to represent the regional loess ridge topography. The seismic input had to be representative of typical seismic activities in the region. This was crucial to ensure the authenticity and applicability of the numerical model.

2. Advanced and Reliable Numerical Software (FLAC3D): FLAC3D is internationally recognized geotechnical engineering analysis software with robust computational capabilities and extensive modeling capabilities. It particularly excels in dynamic analysis. FLAC3D's reliable handling of dynamic boundary conditions and seismic load input has been confirmed and acknowledged in numerous studies. Using a fully nonlinear analysis approach based on the explicit difference method, FLAC3D employs node-centered mass derived from real density in the surrounding region to solve all motion equations. The appropriate setting of lag damping, in terms of damping form and parameters, allows the description of the soil's hysteresis loop and its behavior under dynamic loading. Lag damping is a material-independent damping format that satisfies the Masing two-fold rule. In dynamic calculations, lag damping can fulfill the Masing two-fold rule, constructing hysteresis loops for soil under dynamic loading. It can directly adopt the modulus reduction curve from dynamic tests to represent the dynamic nonlinear characteristics of loess.

Based on these two factors, we conducted borehole exploration in loess ridges to obtain detailed geological and seismic velocity structure characteristics. Intact loess samples obtained from boreholes underwent laboratory tests and resonant column tests. Laboratory tests provided parameters such as density, water content, cohesion, and internal friction angle, essential for static slope stability analysis. Resonant column tests yielded dynamic shear modulus ratios and strain curves at different depths, used to calibrate lag damping parameters. Lag damping was implemented in various forms, and this study adopted a three-parameter form, adjusted through iterative refinement to closely match the results of dynamic tests.

14. Have the results of the numerical models been validated against actual seismic event data? If so, how closely do the model predictions align with real-world observations, and what discrepancies were noted?

Reponse14：In the study area, there is a lack of seismograph networks specifically for loess slope terrain, and there are no actual seismic records for loess slopes to compare with. The aftershock results from the topographic array flow observation set on a loess single-sided slope in Wen County after the 2008 Wenchuan Ms8.0 earthquake show that the PGA at the crest is 1.5-2.0 times that at the base of the slope. However, it should be noted that the site is relatively distant from the epicenter (approximately 240 km), and the slope is a single-sided slope, which provides limited comparability.

15. What are the acknowledged limitations of the generalized numerical model applied in this study? How might these limitations impact the interpretation and application of the findings? Please include these and the relevant discussion in the revised manuscript.

Reponse15：Due to the complexity of loess topography and stratigraphic structures, the selected study area may not represent all aspects of loess ridge landforms and geological characteristics. Geological and soil differences may lead to varied seismic responses. Additionally, the irregular terrain and erosional features of loess ridges can introduce complexities in soil vibration and amplification effects. It is essential to compare these aspects with recorded topography data from the region's terrain arrays to gain a more in-depth understanding of the amplification effects in loess ridge topography.

16. How does the seismic response of loess slopes vary with different levels of seismic intensity? Are there specific intensities where the amplification effects become significantly more pronounced?

Reponse16：Compared to low-intensity seismic motion (0.1 m/s²), the ε-η curves under high-intensity seismic motion at low frequencies (1-2 Hz) exhibit a consistent growth pattern across various models. However, a significant difference is that under strong seismic conditions, ε values tend to be higher in most models, and the ε-η curves transition from a smoother pattern observed during small seismic motions to a more intricate and irregular behavior.

The suggestion to investigate whether there are specific intensity levels of seismic motion that significantly enhance the amplification effects in loess slopes is excellent. We will conduct a dedicated study on this topic.

17. To what extent do variations in soil parameters such as moisture content, density, and composition affect the conclusions drawn from this study? Could the authors elaborate on how these factors were accounted for in their analysis?

Reponse17：The loess samples employed in our study were obtained from an actual loess ridge slope, and their physical and dynamic properties were determined through laboratory experiments. Considering that varying initial overburden pressure, porosity, and moisture content characterize the loess at different depths, the dynamic characteristics of the loess differ accordingly. Based on the shear wave velocity structure and physical parameters, the loess ridge was divided into four layers. The layering approach considers the variations in parameters such as moisture content, overburden pressure, and porosity at different depths of the loess. In this region, the differences in the thickness of the loess cover are not significant, ensuring representativeness.

18. How do factors of uncertainty in seismic motion (such as unpredictable intensity and duration) affect the conclusions of the study? Were these uncertainties considered in the modeling process?

Reponse18：The model primarily considered two seismic motion intensities and frequency characteristics. A typical seismic signal for loess terrain was employed without further consideration of features such as intensity and duration.

19. What areas for future research do the authors identify based on their findings? Are there specific aspects of loess slope seismic behavior that require further investigation?

Reponse19：The research findings presented in this paper offer a scientific basis for seismic input considerations in the seismic design of engineering structures in the Loess Plateau region. Considering the recommendations and proposed research directions from the reviewer, we identify the following areas for further exploration:

Investigation of Seismic Terrain Effects in the Loess Plateau Region: Establishing representative slope models that capture the diverse geographical and topographical features of the Loess Plateau. Conducting a detailed study on seismic terrain effects under varying seismic characteristics, such as intensity and duration, will provide valuable insights for seismic disaster prevention and mitigation in the region.

Analysis of Parameter Sensitivity: addressing the reviewer's suggestion regarding a sensitivity analysis of key parameters to assess their impact on seismic response. Identifying the most sensitive parameters in the model will guide future research in refining the understanding of these influential factors.

Coupled Interactions between Elements and Terrain Effects: investigating the soil-structure interaction of buildings situated on slope surfaces and examining their influence on seismic motion and slope stability. Additionally, exploring how changes in slope features, including erosion, impact seismic motion and slope stability. Delving into these aspects will significantly enhance the depth and practical relevance of the study.

20 & 21 please make sure you use proper superscripts, etc in units and others, for example in table 1 (kg/m3). "3" should be a superscript. fonts are not properly scaled in figures. see for example fig 4 where the fonts used for the axes of the graph are very small and can hardly be read. the same for fig 5 and others. fonts should be properly scaled. in fact most figures have this problem, I think. Please update ALL figures with proper fonts that can be read either on the screen or on the paper. the same for table 2 where again fonts are too small, but this can be fixed by the journal later on.

Reponse20&21：Thank you for pointing out these shortcomings. The unclear and improperly formatted figures have been updated.
